# Micafungin for the treatment of proven and suspected invasive candidiasis in children and adults: findings from a multicenter prospective observational study

**DOI:** 10.1186/s12879-014-0725-7

**Published:** 2014-12-31

**Authors:** Claudio Viscoli, Matteo Bassetti, Elio Castagnola, Simone Cesaro, Francesco Menichetti, Sandra Ratto, Carlo Tascini, Daniele Roberto Giacobbe

**Affiliations:** Infectious Diseases Unit, IRCCS San Martino University Hospital – IST, University of Genoa, L.go R. Benzi, Genoa, 10-16132 Italy; Infectious Diseases Division, Santa Maria Misericordia Hospital, Piazzale Santa Maria della Misericordia, Udine, 15-33100 Italy; Infectious Disease Unit, Istituto Giannina Gaslini, Largo G.Gaslini, Genoa, 5-16147 Italy; Paediatric Hematology Oncology, Azienda Ospedaliera Universitaria Integrata, Verona, P.le L.A. Scuro, Verona, 10-37134 Italy; U.O.C. Malattie Infettive, Azienda Ospedaliera Universitaria Pisana, Via Paradisa, Cisanello, 2 – 56100 Pisa, Italy

**Keywords:** Micafungin, Safety, Clinical response, Invasive candidiasis, Candidemia, Post marketing surveillance

## Abstract

**Background:**

A multicenter observational study was conducted in Italy to assess the safety of micafungin in the daily clinical practice for the treatment of proven and suspected invasive candidiasis (IC), as well as to describe rates of clinical response to micafungin treatment.

**Methods:**

From October 2010 to March 2012, data from consecutive eligible neonate, pediatric, and adult patients treated with micafungin for a proven or suspected IC were collected. Patients were deemed as eligible if they or their parents signed an informed consent. The study endpoints were to assess safety of micafungin in the treatment of both proven and suspected IC, and to describe rates of clinical response to micafungin treatment. Clinical response was assessed at the end of micafungin treatment (EOMT) and defined as favorable (complete or partial resolution of signs and symptoms) or unfavorable (stability or progression).

**Results:**

During the study period, 108 patients with proven or suspected IC were enrolled. Thirty-six out of 108 patients (33%) were < 18 year-old (median 1 year), whereas 72 (67%) were ≥ 18 year-old (median 71 years). Neonates in NICU accounted for 36% of pediatric patients, with the majority of them (54%) being extremely low birth weight (ELBW) newborns. Fifty-eight out of 108 patients (54%) received micafungin for a proven IC, whereas 50/108 patients (46%) were treated for a suspected IC. Among proven IC, candidemia accounted for the majority of events (54/58, 93%), with *Candida albicans* (35/58, 60%) as the most frequently isolated species. Therapy was discontinued due to occurrence of an adverse event in 4/108 subjects (4%). No pediatric patient had treatment interruption because of adverse events. A 67% favorable response rate was observed at EOMT. No age-, species-, underlying conditions- or ward-related differences of favorable response were observed. Survival at EOMT was 90% (97/108 patients), with rates of 97% (35/36) and 86% (62/72) among children and adults, respectively.

**Conclusions:**

Micafungin was well tolerated in a heterogeneous real world population with a bimodal age distribution. A high rate of favorable response to micafungin treatment was reported in both proven and suspected IC.

**Electronic supplementary material:**

The online version of this article (doi:10.1186/s12879-014-0725-7) contains supplementary material, which is available to authorized users.

## Background

Candidemia and other forms of invasive candidiasis (IC) are a major cause of morbidity and mortality [1]–[7]. Incidence of IC is considerably elevated among the extremes of age, with high rates observed both in the elderly and in premature newborns in neonatal intensive care unit (NICU) [8]–[10]. Therefore, safe and effective treatment options are needed to deal with IC in the heterogeneous setting of the daily clinical practice.

Recent guidelines for the management of IC from the European Society of Clinical Microbiology and Infectious Diseases (ESCMID) recommend an echinocandin as the first-line treatment in IC, due to the availability of a large amount of efficacy and safety data [11]–[13].

Micafungin received approval from the major regulatory agencies in the world (e.g., the Food and Drug Administration, FDA, and the European Medicines Agency, EMA) for the treatment of IC in adult and pediatric patients including premature newborns [14]. Clinical trials involving micafungin showed response rates ranging from 71% to 90% in the treatment of IC, and micafungin was well tolerated and safe [15]–[18]. However, tolerability of this drug in the daily clinical experience may vary from that observed in randomized studies, due to the heterogeneous health background of patients in the real world practice. Therefore, a continuous post-marketing evaluation in the real world practice is necessary to adequately validate the safety of micafungin. Aim of the present study was to prospectively evaluate safety of micafungin for the treatment of proven and suspected IC in different clinical conditions and centers, as well as to describe rates of clinical response to micafungin treatment.

## Methods

From October 2010 to March 2012 a multicenter prospective observational study (i.e. a post marketing multicenter surveillance) was conducted in 38 Italian Centers. All investigators decided the use of micafungin in any given patient according to his/her clinical judgment and not according to a specific protocol. They were simply asked to record indications for the use, outcome and tolerability. Institutional Review Boards and/or Independent Ethics Committees at each participating center approved the study protocol (Additional file 1: Table S1), and patients or their parents signed an informed consent for the collection of the data needed for the study.

### Patient population and IC definitions

No age or other protocol-defined exclusion criteria were defined and all patients receiving micafungin for a proven or suspected IC were deemed as eligible, if they accepted to sign an informed consent.

Proven IC was defined as recovery of *Candida* spp. from a normally sterile site in presence of signs and symptoms of infection, according to the European Organization for Research and Treatment of Cancer/National Institute of Allergy and Infectious Diseases Mycoses Study Group (EORTC/MSG) [19]. Suspected IC were diagnosed by investigators according to their clinical judgment, in presence of risk factors for IC and clinical, radiological or laboratory abnormalities (e.g., colonization, β-D-glucan) consistent with an infectious disease process.

### Study design

Consecutive eligible patients receiving micafungin for the treatment of a proven or suspected IC were enrolled. Safety data were collected from adult, pediatric and neonatal patients hospitalized in NICU, ICU, medical and surgical wards. In addition, baseline demographic and clinical variables, including microbiological and laboratory data, were recorded and response to treatment was assessed by the investigators at the end of micafungin treatment (EOMT).

Endpoints of the study were to evaluate tolerability of micafungin in the treatment of proven or suspected IC, and to describe rates of clinical response to micafungin treatment.

Safety data was registered by the investigators and all serious and non-serious events related to the administration of micafungin were reported. Adverse events that were life-threatening, required initial or prolonged hospitalization, or resulted in death, incapacity, or birth defects were considered as serious adverse events, along with any other event deemed as clinically significant by investigators.

The type of response to micafungin treatment was recorded according to investigators’ judgment. A favorable response was defined as complete or partial resolution of signs and symptoms of IC, whereas treatment failure was defined as death from any cause, progression of the disease, or stability of attributable signs and symptoms, reflecting standard definitions [20]. Survival was assessed at the EOMT and at 14 days after EOMT.

Patients were treated according to local standard practice and no approved or investigational medications were provided to the participating centers, reflecting the observational nature of the study.

### Statistical analysis

Safety data, along with demographic and clinical characteristics of patients, was provided with summary statistics. Numbers and percentage of treatment discontinuation because of adverse events and changes in liver enzymes and bilirubin values during treatment were also summarized.

Rates of response at the EOMT were provided for patients’ subgroups according to age, certainty of IC, type of therapy (monotherapy vs. combined therapy), *Candida* spp. responsible for proven IC, recent surgery, presence of active malignancy, allogeneic HSCT, neutropenia at baseline, and ward where the diagnosis of IC was made. Results were then compared using *χ*2 test or Fisher exact test, as appropriate. All tests were 2-sided. Relative risks (RR) with their 95% confidence intervals (CI) were provided. Finally, summary statistics were provided for survival at EOMT and at 14 days after EOMT.

## Results

During the study period, 109 patients were considered as eligible. However, two patients (2%) were excluded from the analyses because they did not receive any antifungal treatment, and one patient was included twice in the study, reflecting two independent IC events. A bimodal age distribution was observed among the 108 definitively enrolled patients, with 36 (33%) patients being < 18 year-old (median 1 year, IQR 1–8) and 72 (67%) ≥ 18 year-old (median 71 years, IQR 62–77). Male and female subjects were 59% (64/108) and 41% (44/108) of the entire population, with no differences in gender distribution between pediatric and adult subjects. Neonates in NICU accounted for 36% of pediatric patients (13/36), they had a median weight of 1000 g (IQR 700–1500) and the majority of them (7/13, 54%) were extremely low birth weight (ELBW, < 1000 g at birth) newborns. Demographic characteristics of patients, along with distributions of wards of hospitalization and underlying conditions, are outlined in Table 1.

**Table 1:** **Baseline characteristics of patients**

Variables	Patients n (%)		
	< 18 years	≥ 18 years	Total
	36 (33)	72 (67)	108 (100)
Age, median (IQR)	1 (0;8)	71 (62;77)	62 (8;74)
Gender, n (%)			
Male	22 (61)	42 (58)	64 (59)
Female	14 (39)	30 (42)	44 (41)
Race, n (%)			
Caucasian	35 (97)	71 (99)	106 (98)
Black	1 (3)	1 (1)	2(2)
Ward of hospitalization, n (%)			
Hematology*	3 (8)	4 (6)	7 (7)
Infectious diseases	1 (3)	31 (43)	32 (30)
Other medical wards**	12 (33)	10 (14)	22 (20)
Surgical	1 (3)	14 (19)	15 (14)
ICU	6 (17)	13 (18)	19 (18)
NICU	13 (36)	-	13 (12)
Underlying conditions and devices, n (%)^†^			
Hematological malignancy	9 (25)	5 (7)	14 (13)
Solid tumor	0	13 (18)	13 (12)
Allogeneic HSCT	5 (14)	0	5 (5)
Diabetes	0	3 (4)	3 (3)
Recent Surgery	3 (8)	19 (26)	22 (20)
Presence of CVC	35 (97)	50 (69)	85 (79)
Neutropenia (missing = 6)	11 (31)	6 (9)	17 (17)

IQR, interquartile range; ICU, intensive care unit; NICU, neonatal intensive care unit; HSCT, hematopoietic stem cell transplantation; CVC, central venous catheter.

*Including HSCT centers.

^†^Frequencies not mutually exclusive.

**Pediatrics (12), gastroenterology (4), nephrology (2), internal medicine (1), oncology (1), neurology (1), pulmonology (1).

Micafungin was administered for a median of 13 days (IQR 8–19 days), with median daily dose of 2.2 mg/kg (IQR 2–4.1 mg/kg) and 100 mg (IQR 100–100 mg) in children and adults, respectively. Micafungin monotherapy was administered to 98/108 patients (91%), whereas 10/108 subjects (9%) received combined antifungal therapy. Antifungals administered with micafungin in combined regimens included lipid formulations of amphotericin B (8/10, 80%), voriconazole (1/10, 10%), and fluconazole (1/10, 10%).

Fifty-eight out of 108 patients (54%) received micafungin for a proven IC, whereas 50/108 patients (46%) were treated for a suspected IC. Among proven IC, candidemia accounted for the majority of events (54/58, 93%), followed by endocarditis (3/58, 5%) and intra-abdominal infections (1/58, 2%). Overall, *Candida albicans* (35/58, 60%) was the most frequently isolated species, followed by *Candida glabrata* (8/58, 14%) and *Candida parapsilosis* (8/58, 14%). Indeed, *C. albicans* was the most frequently isolated species in adults (29/45, 64%) and children (4/6, 67%), whereas *C. parapsilosis* was the prevalent causative agent of proven IC among neonates in NICU (4/7, 57%). Details of reported IC, along with microbiological data, are outlined in Table 2.

**Table 2:** **Micafungin for the treatment of IC in daily clinical practice**

Type of IC	Patients n (%)		
	< 18 years	≥ 18 years	Total
	36 (33)	72 (67)	108 (100)
Proven IC*, n	13	45	58
Candidemia	13	41	54
Endocarditis	-	3	3
Intra-abdominal infections	-	1	1
Suspected IC, n	23	27	50
Diagnostic-driven therapy^†^	5	4	9
Empirical therapy	18	23	41

IC, invasive candidiasis.

Intra-abdominal infection was defined as *Candida* spp. isolation from peritoneal fluid with clinical signs of invasive infection, according to investigator’s clinical judgment.

*Proven IC.

Neonates (n = 7): *C. parapsilosis* (n = 4), *C. albicans* (n = 2), *C. famata* (n = 1).

Children (n = 6): *C. albicans* (n = 4), *C. parapsilosis (n =* 1), *C. glabrata* (n = 1).

Adults (n = 45): *C. albicans* (n = 28), *C. parapsilosis* (n = 3), *C. glabrata* (n = 6), *C. tropicalis* (n = 2), *C. albicans* + *C. glabrata* (n = 1), *C. guilliermondii* (n = 1), *C. krusei* (n = 1), *C.krusei + C. lusitanie* (n = 1), *C. sake* (n = 1), *C. kefir* (n = 1).

^†^According to mannan antigen and/or (1,3)-β-D-Glucan, which resulted positive in 1/2 and 9/13 tested patients, respectively.

### Safety

Overall, 5 adverse drug reactions to micafungin were reported during the study period: 4 were classified as non-serious (anemia, rash, increasing bilirubin and exacerbation of pre-existing tremor) and 1 was serious (increase of liver enzymes). Micafungin administration was interrupted in 12/108 patients (11%). Therapy was discontinued due to reported ineffectiveness in 8/12 and occurrence of an adverse event in 4/12 patients, 4% of all treated subjects. Adverse drug reactions leading to treatment discontinuation were anemia, rash, increasing bilirubin in a patient with pre-existing liver disease due to chronic HCV-related hepatitis and primary biliary cirrhosis, and severe hepatic failure, defined by an increase in liver enzymes plasmatic values with a concomitant reduction of prothrombin time. One patient developed an exacerbation of a pre-existing tremor, but micafungin was not discontinued and the patient completed the treatment without any major trouble. No pediatric patient had treatment interruption because of adverse drug reactions.

An increase (>2.5-fold the upper limit of normal) of aspartate aminotransferase (AST), alanine aminotransferase (ALT), and bilirubin was observed in 5/60 (8%), 5/61 (8%), and 1/41 (2%) patients for whom values at EOMT were available, respectively. In 23/95 patients (24%, missing = 13) transaminases were higher than normal values at the beginning of micafungin administration, and in 8/23 of them (35%) they returned within the normal range during therapy.

According to investigators’ opinion, no case of death in this study was attributed to micafungin administration.

### Response to treatment and survival rates

A favorable response was observed in 70/104 patients (67%, missing = 4). Complete and partial response was reported for 52/70 (74%) and 18/70 (26%) patients, respectively. No age-, species-, underlying conditions- or ward-related differences of response were observed. However, although not statistically significant, response was slightly lower in patients ≥ 18 years (RR 0.8, 95% CI 0.6-1.1, p = 0.14). Rates of response according to different subgroups are summarized in Table 3.

**Table 3:** **Favorable response to micafungin therapy in different subgroups**

Subgroups	Favorable response %, (n)	Relative Risk (95% CI)	p
Total			
(n = 104/108, missing = 4)	67 (70/104)		
Type of IC			0.77
Proven IC	66 (37/56)	1 (ref)	
Suspected IC	69 (33/48)	1.04 (0.80 - 1.36)	
Therapy			0.32
Monotherapy	69 (65/94)	1 (ref)	
Combined therapy^†^	50 (5/10)	0.72 (0.38 - 1.36)	
Age			0.14
Patients < 18 years	76 (26/34)	1 (ref)	
Patients ≥ 18 years	63 (44/70)	0.82 (0.63 - 1.06)	
Pathogen			0.76
*C. albicans*	68 (23/34)	1 (ref)	
Non-albicans *Candida* spp.*	64 (14/22)	0.94 (0.64 - 1.39)	
Recent surgery			0.47
Yes	60 (12/20)	1 (ref)	
No	69 (58/84)	1.15 (0.78 - 1.69)	
Neutropenia (missing = 6)			0.38
ANC < 500/mm^3^	76 (13/17)	1 (ref)	
ANC ≥ 500/mm^3^	67 (54/81)	0.87 (0.64 - 1.18)	
Active malignancy			0.66
Yes**	71 (17/24)	1 (ref)	
No	66 (53/80)	0.95 (0.69 - 1.26)	
Allogeneic HSCT			0.75
Yes	60 (3/5)	1 (ref)	
No	68 (67/99)	1.13 (0.54 - 2.34)	
Ward			0.18
ICU/NICU	57 (17/30)	1 (ref)	
Non-ICU/NICU	72 (53/74)	1.26 (0.89 - 1.78)	

IC, invasive candidiasis; HSCT, hematopoietic stem cell transplantation; ICU, intensive care unit; NICU, neonatal intensive care unit; ANC, absolute neutrophil count.

^†^lipid formulations of amphotericin B (8/10, 80%), voriconazole (1/10, 10%), and fluconazole (1/10, 10%).

*C. parapsilosis 6/8 (75%), *C. glabrata* 4/7 (57%), other *Candida* spp. or polymicrobial 4/7 (57%).

**Hematological (n = 9) or solid (n = 8) neoplasms.

Reported survival at EOMT was as high as 90% (97/108 patients), with rates of 97% (35/36) and 86% (62/72) among < 18 and ≥ 18 year-old subjects, respectively. Data at 14 days after EOMT was available for 81/108 patients (75%, missing = 27), and an overall survival of 73% was reported (59/81). Similarly to results at EOMT, a higher survival at 14 days after EOMT was observed for pediatric patients (93%, 26/28) in comparison with adult subjects (62%, 33/53). It is worth noting that survival at 14 days after EOMT was probably underestimated, since missing data mostly refers to patients who early and fully recovered from IC, and that were thus discharged home before survival assessment at 14 days after EOMT.

## Discussion

The use of micafungin for the treatment of proven and suspected IC was assessed in a multicenter prospective and observational study performed in a real world population with a bimodal age distribution. This type of study, i.e. a post marketing surveillance, has the major aim in registering adverse events in the everyday clinical practice [21]. In this study, micafungin was well tolerated, with only 4% of withdrawal because of adverse drug reactions.

Transient increases in aminotransferases are common in safety reports regarding all echinocandins [22]–[24]. In the present study, increases in transaminases values during micafungin treatment were consistent with those observed in randomized clinical trials and lower than those observed with other antifungals such as amphotericin B lipid formulations and voriconazole [25]. We observed only a case of hepatic failure leading to treatment discontinuation, occurring in a critically ill 73-year-old patient with multiple comorbidities and receiving other potentially hepatotoxic drugs, including amiodarone and paracetamol, that obfuscate any potential role for micafungin. We thus confirmed the already existent data of limited hepatic toxicity after short-term micafungin exposure. However, micafungin hepatic toxicity is a concern that has led to an EMA warning about possible risks of hepatic tumors, as oncogenicity was observed in animal studies in which micafungin was used at incomparably higher dosages than those administered to humans, and for a very long time [26]. Although we understand that this warning may pose some concerns and confusion in the medical community at least in Europe, at recommended and approved dosages, oncogenicity was not reproduced in clinical trials, and micafungin has been used worldwide in more than one million patients, with no post-marketing reports of hepatic carcinomas or adenomas related to micafungin treatment (Astellas Global Safety Database, October 2012, data on file). In addition, the presumptive association of catechol metabolism (one of micafungin metabolites [M1] carries a catechol group in its molecule) and development of hepatocellular cancer has not been confirmed, and short-term exposures consistent with clinical use did not alter the rate of cancer cell growth in a human *ex vivo* liver model [27]. Finally, it is unknown whether or not long-term and high dose studies have been performed for other echinocandins as well.

In this study, we observed rates of response to micafungin treatment that were slightly lower than those reported in randomized studies [15]–[18]. Indeed, the 63% favorable response observed in adult patients in our study was to some extent lower than those of 71-90% reported in clinical trials [15]–[17]. However, demographic variables and distribution of underlying conditions in the real life setting are expected to vary from those of strictly selected patients in randomized studies (e.g., exclusion of subjects with reduced life expectancy, severe organ impairment and/or receiving concomitant medications), and might explain the differences in terms of response observed in our study. As regards pediatrics, the rate of favorable response was 76%, consistent with that reported by the largest available clinical trial [18]. Of note, observed survival rates in both adults and children were comparable with those reported in clinical trials and in multicenter international surveillance studies [15]–[18],[28],[29].

Several limitations of this study have to be acknowledged. First of all, some eligible patients may have refused to sign an informed consent. Since the number of patients refusing enrollment was not registered by investigators, we could not weigh the intrinsic selection bias related to this variable. Second, the follow-up after EOMT was reduced in comparison to clinical trials, and possible toxicities and deaths occurring later than two weeks after EOMT could not be reported. Finally, we also enrolled patients with suspected IC for safety evaluation, in line with the study major aim. Therefore, an inaccurate classification of disease could have occurred in some of these cases. This possibility, along with the absence of a comparator arm, prevented from an appropriate analysis of micafungin effectiveness. However, this was not a comparative study and our primary purpose was to describe the safety of micafungin among patients with both proven and suspected infections in the daily clinical practice. Rates of response were provided only as complementary informative data. Therefore, any interpretation about micafungin effectiveness in this study should be weighed carefully and must consider all the aforementioned limitations.

## Conclusions

In the present study, we observed a very low rate of adverse events in patients with proven or suspected IC treated with micafungin. Micafungin resulted well tolerated in a heterogeneous real world population with a bimodal age distribution. A high rate of favorable response to micafungin treatment was reported in both proven and suspected infections.

## Additional file

## Electronic supplementary material

Additional file 1: Table S1.: List of Independent Ethics Committees and/or Institutional Review Boards that approved the study protocol. (PDF 158 KB)
