# Supplementary material for: Micafungin for the treatment of proven and suspected invasive candidiasis in children and adults: findings from a multicenter prospective observational study
Source: BMC Infect Dis. 2014 Dec 31;14:725. doi: 10.1186/s12879-014-0725-7 (PMC4297391; doi:10.1186/s12879-014-0725-7)
Supplement: Supplementary file 1 — Additional file 1: Table S1.: List of Independent Ethics Committees and/or Institutional Review Boards that approved the study protocol. (PDF 158 KB) [file 12879_2014_725_MOESM1_ESM.pdf]

Table S1. List of Independent Ethics Committees and/or Institutional Review Boards that approved the study protocol.

**Center**	**Independent Ethics Committee and/or Institutional Review Board**
Clinica di Malattie Infettive<br><br>Azienda Ospedaliera Universitaria San Martino<br><br>Genova, Italy	**COMITATO ETICO DELL´AZIENDA OSPEDALIERA UNIVERSITARIA S. MARTINO DI GENOVA**
Unità Operativa Complessa Malattie Infettive<br><br>Istituto Scientifico. G. Gaslini<br><br>Genova, Italy	**COMITATO DI ETICA DELL'IRCCS ISTITUTO GIANNINA GASLINI DI GENOVA**
U.O. Malattie Infettive<br><br>Ospedale S. Andrea<br><br>Vercelli, Italy	**COMITATO ETICO DELL´A.O.U.**<br><br>**MAGGIORE DELLA CARITA´ DI NOVARA**
S.C. Malattie Infettive e Tropicali<br><br>Ospedale S. Croce<br><br>Cuneo, Italy	**COMITATO ETICO DELL´A.O. S. CROCE E CARLE DI CUNEO**
U.O. Malattie Infettive<br><br>Ospedale S. Paolo<br><br>Savona, Italy	**COMITATO ETICO AZIENDALE DELLA ASL 2 SAVONESE DI SAVONA**

II Divisione Malattie Infettive<br><br>Ospedale L. Sacco<br><br>Milano, Italy	**COMITATO ETICO LOCALE PER LA SPERIMENTAZIONE CLINICA DELL'AZIENDA OSPEDALIERA LUIGI SACCO DI MILANO**
U.O. Malattie Infettive e Tropicali<br><br>Ospedale Circolo e Fondazione Macchi<br><br>Varese, Italy	**COMITATO ETICO DELL´AZIENDA OSPEDALIERA OSPEDALE DI CIRCOLO E FONDAZIONE MACCHI DI VARESE**
Oncoematologia pediatrica e trapianto di midollo<br><br>A.P. Spedali Civili di Brescia<br><br>Brescia, Italy	**COMITATO ETICO DELL´AZIENDA OSPEDALIERA SPEDALI CIVILI DI BRESCIA**
U. O. Malattie Infettive<br><br>Ospedale Cisanello<br><br>Pisa, Italy	**CE PER LA SPERIMENTAZIONE CLINICA DEI MEDICINALI DELL'AZIENDA OSPEDALIERO UNIVERSITARIA PISANA DI PISA**
U.O.C. Terapia Intensiva Neonatale<br><br>A.O.U.S. Dipartimento di Pediatria, Ostetricia e Medicina della Riproduzione<br><br>Università degli Studi di Siena<br><br>Siena, Italy	**COMITATO ETICO LOCALE PER LA SPERIMENTAZIONE CLINICA DEI MEDICINALI DELL´AZIENDA OSPEDALIERA UNIVERSITARIA SENESE DI SIENA**
Anestesia e Rianimazione<br><br>Ospedale nuovo S. Giovanni di Dio<br><br>Firenze, Italy	**COMITATO ETICO PER LA SPERIMENTAZIONE CLINICA DEI MEDICINALI DELL'AZIENDA SANITARIA DI FIRENZE**

S.O.D. Malattie Infettive e Tropicali<br><br>A. Ospedaliero-Universitaria Careggi<br><br>Firenze, Italy	**COMITATO ETICO PER LA SPERIMENTAZIONE CLINICA DEI MEDICINALI DELL´AZIENDA OSPEDALIERO-UNIVERSITARIA CAREGGI DI FIRENZE**
S.O.D. Malattie infettive<br><br>A. Ospedaliero-Universitaria Careggi<br><br>Firenze, Italy	**COMITATO ETICO PER LA SPERIMENTAZIONE CLINICA DEI MEDICINALI DELL´AZIENDA OSPEDALIERO-UNIVERSITARIA CAREGGI DI FIRENZE**
Malattie Infettive 1<br><br>Fondazione ICCRS Policlinico S. Matteo<br><br>Pavia, Italy	**COMITATO DI BIOETICA DELLA FONDAZIONE IRCCS POLICLINICO S. MATTEO DI PAVIA**
U.O. di Malattie Infettive<br><br>A. O. Ospedale di Circolo di Busto Arsizio<br><br>Busto Arsizio, Italy	**COMITATO ETICO DELL´AZIENDA OSPEDALIERA OSPEDALE DI CIRCOLO DI BUSTO ARSIZIO (VA)**
Oncoematologia Pediatrica<br><br>Ospedale Policlinico G.B. Rossi<br><br>Verona, Italy	**COMITATO ETICO PER LA SPERIMENTAZIONE DELL´AZIENDA OSPEDALIERA ISTITUTI OSPITALIERI DI VERONA**
U.O. Malattie Infettive e Tropicali<br><br>Ospedale Civile di Pescara<br><br>Pescara, Italy	**COMITATO ETICO PER LA SPERIMENTAZIONE CLINICA DEI FARMACI DELLA USL DI PESCARA**

Oncoematologia Pediatrica<br><br>Azienda Ospedaliera di Perugia<br><br>Ospedale S Maria della Misericordia<br><br>Perugia, Italy	**COMITATO ETICO DELLE AZIENDE SANITARIE DELL´UMBRIA DI PERUGIA**
Clinica di Malattie Infettive Università Politecnica delle Marche<br><br>Torrette di Ancona, Italy	**COMITATO ETICO DELL' AZIENDA OSPEDALIERA UNIVERSITARIA "OSPEDALI RIUNITI UMBERTO I- G.M. LANCISI- G. SALESI DI ANCONA**
Malattie Infettive<br><br>Università La Sapienza<br><br>Policlinico Umberto I°<br><br>Roma, Italy	**COMITATO ETICO DELL´AZIENDA POLICLINICO UMBERTO I DI ROMA**
U.O.C. Servizio di Malattie Infettive<br><br>Dipartimento di Medicina<br><br>Università Tor Vergata<br><br>Roma, Italy	**COMITATO ETICO INDIPENDENTE DELL'AZIENDA OSPEDALIERA UNIVERSITARIA POLICLINICO TOR VERGATA DI ROMA (Fondazione PTV)**
Medicina preventiva delle migrazioni del turismo e dermatologia tropicale<br><br>Istituti Fisioterapici Ospitalieri Regina Elena<br><br>Roma, Italy	**COMITATO ETICO DELL'IRCCS ISTITUTI FISIOTERAPICI OSPITALIERI DI ROMA**

U.O. di Immunoinfettivologia Pediatrica<br><br>Ospedale Bambin Gesù<br><br>Roma, Italy	**COMITATO ETICO PER LA SPERIMENTAZIONE CLINICA DELL´ IRCCS OSPEDALE PEDIATRICO BAMBINO GESU´ DI ROMA**
Malattie Infettive<br><br>ASL di Viterbo<br><br>Ospedale Belcolle<br><br>Viterbo, Italy	**COMITATO ETICO DELLA AUSL DI VITERBO**
Ematologia<br><br>IME – Policlinico di Tor Vergata<br><br>Roma, Italy	**COMITATO ETICO INDIPENDENTE DELL’AZIENDA OSPEDALIERA UNIVERSITARIA POLICLINICO TOR VERGATA DI ROMA (Fondazione PTV)**
U. O. Malattie Infettive<br><br>A.O.U. “OO.RR. S. Giovanni di Dio e Ruggi D’Aragona”<br><br>Salerno, Italy	**COMITATO ETICO DELL´AZIENDA OSPEDALIERA OSPEDALI RIUNITI S. GIOVANNI E RUGGI D´ARAGONA DI SALERNO**
Dipartimento di Scienze Chirurgiche, Anestesiol.-Rianimatorie e dell'emergenza<br><br>A. O. Universitaria<br><br>Università degli Studi di Napoli Federico II°<br><br>Napoli, Italy	**COMITATO ETICO PER LE ATTIVITA’´ BIOMEDICHE DELL´UNIVERSITA’ DEGLI STUDI FEDERICO II DI NAPOLI**

U.O. Rianimazione e T.I.<br><br>A. O. Bianchi Melacrino – Morelli<br><br>Reggio Calabria, Italy	**COMITATO ETICO DELL´AZIENDA OSPEDALIERA BIANCHI-MELACRINO-MORELLI DI REGGIO CALABRIA**
Rianimazione<br><br>Ospedale Pugliese – Ciaccio<br><br>Catanzaro, Italy	**COMITATO ETICO DELL´AZIENDA OSPEDALIERA PUGLIESE-CIACCIO DI CATANZARO**
U.O. Malattie Infettive<br><br>A.O. di Cosenza<br><br>P.O. SS Annunziata<br><br>Cosenza, Italy	**COMITATO ETICO DELL´AZIENDA OSPEDALIERA DI COSENZA**
U.O.C. Neonatologia e TIN<br><br>A.O. di Cosenza<br><br>P.O. SS Annunziata<br><br>Cosenza, Italy	**COMITATO ETICO DELL´AZIENDA OSPEDALIERA DI COSENZA**
Ematologia<br><br>Azienda Ospedaliera Cardinale G. Panico<br><br>Tricase, Italy	**COMITATO ETICO DELL´AZIENDA OSPEDALIERA CARDINALE GIOVANNI PANICO DI TRICASE (LE)**

Clinica di Malattie Infettive<br><br>Azienda Ospedaliero-Universitaria<br><br>Ospedali Riuniti<br><br>Foggia, Italy	**COMITATO ETICO DELL´AZIENDA OSPEDALIERO-UNIVERSITARIA OSPEDALI RIUNITI DI FOGGIA**
Dip. Materno-Infantile<br><br>Neonatologia e Terapia Intensiva Neonatale<br><br>Ospedale SS Annunziata<br><br>Chieti, Italy	**COMITATO ETICO DELL'UNIVERSITA' DEGLI STUDI GABRIELE D`ANNUNZIO E DELLA ASL 2 LANCIANO-VASTO-CHIETI DI CHIETI**
U.O. Neonatologia e T.I.N.<br><br>Ospedale Civile di Pescara<br><br>Pescara, Italy	**COMITATO ETICO PER LA SPERIMENTAZIONE CLINICA DEI FARMACI DELLA USL DI PESCARA**
Patologia Neonatale<br><br>P.O. San Giovanni di Dio<br><br>Azienda Ospedaliera-Universitaria di Cagliari<br><br>Cagliari, Italy	**COMITATO ETICO DELL´AZIENDA OSPEDALIERO-UNIVERSITARIA – UNIVERSITA' DI CAGLIARI**
Divisione di Ematologia<br><br>Azienda Sanitaria dell'Alto Adige<br><br>Bolzano, Italy	**COMITATO ETICO DEL COMPRENSORIO SANITARIO DI BOLZANO**

Dipartimento di Medicina Clinica 1<br><br>Divisione Malattie Infettive e Tropicali<br><br>Ospedale dell'Angelo e Ospedale S.S. Giovanni e Paolo<br><br>Venezia, Italy	**COMITATO ETICO PER LE SPERIMENTAZIONI CLINICHE DEI MEDICINALI DELLA PROVINCIA DI VENEZIA**
